# Draft Genome Sequence of USA100 Methicillin-Resistant *Staphylococcus aureus* Strain 209

**DOI:** 10.1128/genomeA.01399-17

**Published:** 2018-01-04

**Authors:** Yingfeng Chen, Heidi A. Crosby, Wilhelm F. Oosthuysen, Daniel J. Diekema, Scott T. Kelley, Alexander R. Horswill

**Affiliations:** a Department of Biology, San Diego State University, San Diego, California, USA; b Department of Immunology & Microbiology, University of Colorado Anschutz Medical Campus, Aurora, Colorado, USA; c Department of Internal Medicine, University of Iowa, Iowa City, Iowa, USA; d Denver VA Healthcare System, Denver, Colorado, USA

## Abstract

USA100 strains are significant contributors to the overall burden of health care-associated methicillin-resistant *Staphylococcus aureus* (MRSA) infections. Strain 209 is a representative MRSA isolate that serves as a model organism for *agr* type II studies and USA100 virulence assessments. We present a draft genome sequence of this strain.

## GENOME ANNOUNCEMENT

USA100 methicillin-resistant *Staphylococcus aureus* (MRSA) isolates represent a major portion of health care-associated MRSA infections. In 2005/2006, USA100 strains caused 53.6% of invasive MRSA infections, more than any other type of MRSA strain (1). In more recent years, this proportion has decreased (2), but USA100 remains a prominent cause of invasive infections, and outcomes for these infections are similar to, if not worse than, those caused by other MRSA strains (3). USA100 strains also typically exhibit a broader spectrum of antibiotic resistance than other isolates (1, 4). There is recent evidence that USA100 strains are moving away from hospitals and into community settings (5), potentially creating new reservoirs for opportunistic infection.

To gain insight into genomic features and virulence traits of USA100 strains, we selected a representative isolate obtained from an MRSA surveillance program at the University of Iowa. Strain 209 (also called IA209) is a throat culture isolate that is *spa* type 002, SCC*mec* type II, Panton-Valentine leukocidin (PVL) negative, and classified by pulsed-field gel electrophoresis as USA100. The strain is resistant to the antibiotics oxacillin, erythromycin, clindamycin, and levofloxacin. Like other USA100s, strain 209 has an *agr* type II system and makes an AIP-II signal (6). *In vitro* testing shows that strain 209 has a robust *agr* system and is a more clinically relevant representative for *agr* type II studies than the historical 502A isolate (7). Strain 209 is able to cause sepsis, infective endocarditis, and pneumonia in rabbit models of infection (8, 9), indicating that the isolate has functional mechanisms of pathogenesis.

To isolate genomic DNA, cells were lysed with lysostaphin and DNA was purified using the Puregene yeast/bacteria kit B (Qiagen). Sequencing libraries were prepared using the Hyper Prep library kit (Kapa Biosystems) and sequenced on an Illumina MiSeq platform using a 250-bp paired-end format, resulting in a total of 5,320,190 reads. The raw sequences were preprocessed using Cutadapt version 1.14 to trim adaptors (10) and using Prinseq version 0.20.4 to perform quality filtering (11). Initial genome assembly was performed with the SPAdes version 3.10.0 genome assembler program (12). Contigs less than 1,000 bp in length and with a coverage depth lower than 10× were excluded, which resulted in 15 contigs with a combined total of 2,769,008 bp (*N*_50_, 324,561 bp) and with a G+C content of 32.72%. The largest contig was 753,539 bp long. Contigs were extended using AlignGraph, an algorithm for secondary *de novo* genome assembly, by aligning contigs and initial raw sequences to closely related references (13). *S. aureus* N315 was identified as the closest related assembled genome, and with this genome AlignGraph was able to extend one contig and combine five other contigs into two contigs. The final scaffolds included 12 contigs with a combined total of 2,773,925 bp and a G+C content of 32.71%. The *N*_50_ value for the genome was 444,128 bp. A total of 2,860 genes were predicted by the NCBI Prokaryotic Genome Annotation Pipeline (14).

### Accession number(s).

The draft genome sequence of strain 209 was deposited at DDBJ/ENA/GenBank under the accession number NTCY00000000.
